# Successful Treatment of Early Endometrial Carcinoma by Local Delivery of Levonorgestrel: A Case Report

**DOI:** 10.1155/2010/431950

**Published:** 2010-10-20

**Authors:** D. Wildemeersch, E. Anderson, K. Lambein, P. Pauwels, M. Dhont

**Affiliations:** ^1^Gynecological Outpatient Clinic and Training Center, Rooseveltlaan 43/44, 9000 Ghent, Belgium; ^2^Woman To Woman Total Care, 3411 North 5th Avenue, AZ 85013, USA; ^3^Goormaghtigh Institute of Pathology, University Hospital Ghent, 9000 Ghent, Belgium; ^4^Department of Obstetrics and Gynecology, University Hospital Ghent, 9000 Ghent, Belgium

## Abstract

We describe a case of a 67-year-old Caucasian woman with an early, moderately-differentiated adenocarcinoma of the endometrium. A levonorgestrel-releasing intrauterine system was inserted, which she tolerated well. A full D&C, following removal of the device, was performed after 9 months, confirming absence of tumoral tissue. Examination after 24 months showed a very thin endometrium, indicating complete remission.

## 1. Introduction

An alternative route for the administration of progestins is via the intrauterine one. Intrauterine progestin delivery, in particular levonorgestrel (LNG), is much more effective in suppressing the endometrium than oral treatment. This is due to the uniform suppression of the endometrium throughout the whole thickness of the mucosa caused by the high tissue concentrations when the hormone is applied locally [1]. This form of treatment should, therefore, be preferred over oral treatment for precancerous lesions of the endometrium and early stage invasive endometrial cancer. Surgery is nowadays still the method of choice for the management of these lesions. However, many patients are not eligible (unfit) for surgery and younger women want to preserve their fertility. For these reasons, local delivery of a potent progestin should be the alternative method of choice in these women if it could be shown to be effective. An intrauterine system also provides optimal patient compliance.

There are only a few reports in the literature which demonstrate successful treatment of early stage endometrial carcinoma with the LNG-releasing intrauterine system (IUS) [2–5]. We present a case of a 67-year-old woman with a moderately differentiated invasive adenocarcinoma of the endometrium who was successfully treated with the LNG-IUS.

## 2. Case Presentation

**Figure 1:**
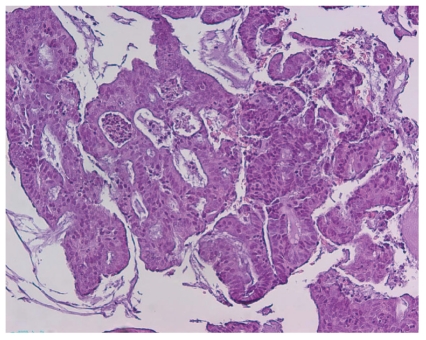
Endometrial curetting prior to therapy, irregular cribriform glands, and mild atypia: well-differentiated endometrioid carcinoma (H&E 100x).

**Figure 2:**
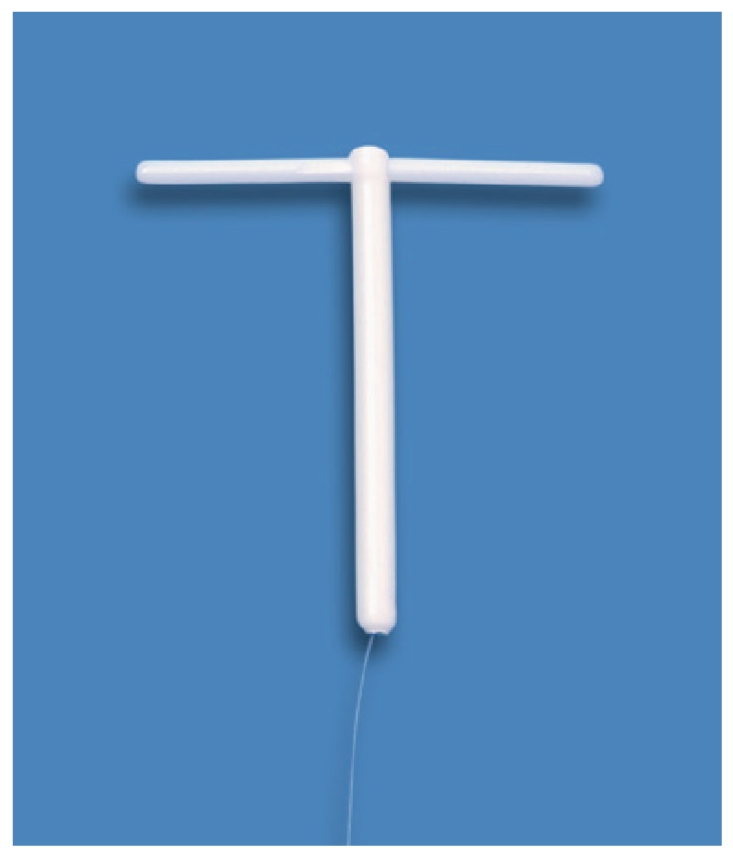
T-shaped Femilis LNG-IUS with 28 mm long crossarm and 3 cm long and 2.4 mm wide drug delivery compartment.

**Figure 3:**
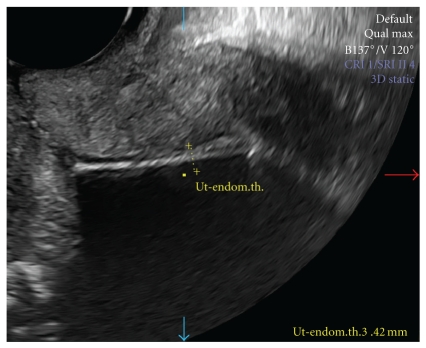
Sonographic view of uterus with LNG-IUS in situ. The endometrial thickness is 3.42 mm.

**Figure 4:**
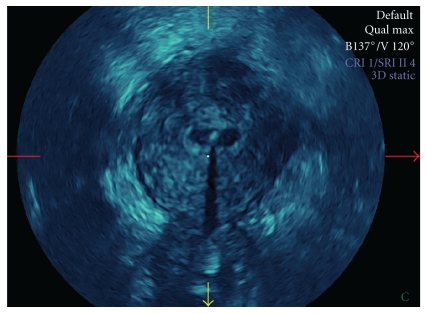
3D-sonographic view of normal uterus (transverse section) with LNG-IUS in center.

**Figure 5:**
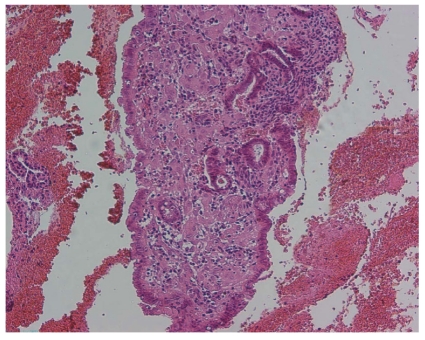
Endometrial curetting posttherapy, small regular glands with tubal metaplasia, surrounded by decidualised stroma (H&E 100x).

In May 2008, a 67-year-old woman residing in the United States of America presented with a history of postmenopausal bleeding since January 2008. She had been using local estrogen treatment for vaginal dryness without any systemic hormonal therapy. She had been diagnosed with an endometrial carcinoma and was advised to undergo surgery. As she wanted to avoid surgery, she sought an alternative treatment and consulted DW. The clinical examination was normal, with normal body weight, and a transvaginal ultrasound scan showed normal appearance of the myometrium and endometrium. An outpatient endometrial pipelle biopsy was performed which revealed a moderately differentiated adenocarcinoma (Figure 1) with minimal myometrial invasion (Clinical FIGO stage I). An LNG-IUS (Femilis, Contrel Research, Ghent, Belgium) releasing 20 *μ*g of LNG/d was inserted (Figure 2), and advice was given to request a repeat biopsy within the next 3 to 6 months. Spotting continued for several weeks and then stopped. As the patient was completely free of symptoms 6 months after insertion of the LNG-IUS, a pelvic transvaginal ultrasound was performed, including a 3D-ultrasound and a Duplex Doppler with Color Flow Mapping. The uterus appeared completely normal, and there was no evidence of any pathology. The endometrium showed normal thickness, and there was no evidence of any endometrial abnormality or myometrial invasion (Figures 3 and 4). The LNG-IUS was identified in situ, as expected. Three months later, in order to ascertain complete remission, the LNG-IUS was removed, and a full D&C was performed. The uterine sound length was 6 cm. The whole cavity was explored, and very scant tissue was removed. Histological examination of the specimen revealed a secretory endometrium without signs of hyperplasia or atypia (Figure 5). A mini Femilis LNG-IUS (crossarm 24 mm long and 28 mm long and 2.0 wide drug compartment) was inserted as a precaution. 

The patient was again examined two years following initial treatment. She had no symptoms, and vaginal ultrasound showed a very thin endometrium and normal position of the LNG-IUS in the uterine cavity.

## 3. Comment

We previously reported on the successful management of nonatypical and atypical endometrial hyperplasia with an LNG-IUS [6, 7]. Endometrial cancer is a very common disorder. Over 40,000 cases are diagnosed in the United States annually with almost 75% of cases presenting with FIGO stage I disease (Cancer fact & figures, 2004). The underlying mechanism for the development of some types of endometrial cancer is exogenous or endogenous hormone stimulation [8]. Therefore, progesterone or progestin treatment, by counteracting the effect of estrogen, may reverse the neoplastic process. Levonorgestrel, delivered locally with a drug delivery system, is probably the best choice as the tissue concentrations are many times higher than when the hormone is administered orally. “Progestasert,” a progesterone-releasing IUS (Alza Corp., Palo Alto, CA), has been used with good results. However, a levonorgestrel-releasing IUS should be preferred as it is much more potent [9]. Histological regression of early endometrial cancer has also been obtained with orally administered progestins but the results cannot be expected to be as good as local treatment [10, 11]. In addition, with an IUS there is less risk of systemic side effects, often leading to poor compliance. Intrauterine delivery of levonorgestrel causes a more uniform response throughout the whole thickness of the endometrium, including the basal layer, compared to intrauterine progesterone delivery or orally administered progestins [12]. The Femilis, currently still an experimental IUS, releases the same amount of LNG as Mirena (BayerSchering Inc., Berlin, Germany) but the initial release is approximately three times higher than the release of LNG of Mirena during the first weeks. This could be advantageous as it increases the impact on the neoplastic endometrial cells. It is possible that the fast remission in this patient is due to the strong suppression of the endometrium immediately after insertion of the LNG-IUS. Several cases of endometrial carcinoma treated with an LNG-IUS (Mirena) have been published [11, 13, 14]. The LNG-IUS should be high up in the fundus to guarantee proper release of the hormone in the fundal area of the uterus. We question if the LNG-IUS was properly located in the uterine cavity in some of these unsuccessful cases. A frameless LNG-IUS should be preferred in case of displacement, partial or total expulsion of a framed LNG-IUS. This LNG-IUS is anchored to the uterine fundus and is unlikely to become displaced or expelled [15]. We would also recommend to remain cautious in women presenting with irregular bleeding after 6 to 12 months following the insertion of an LNG-IUS and to evaluate the endometrium thoroughly. 

Evidently, the best candidates for local treatment with the LNG-IUS are those with no or only minimal endometrial invasion. In these cases, the LNG-IUS could be the treatment of choice especially in women with contraindications for surgery, or even for older women. We agree with Bahamondes et al. that the intrauterine route should be preferred over the oral route. Perhaps, the LNG-IUS, releasing 20 *μ*g of LNG/d, or a higher dose, may be curative in a larger proportion of women with early endometrial carcinoma. Clearly further studies are needed to elucidate this possibility. 

In the meantime, we can conclude that selected women could benefit from treatment with LNG-IUS but we should keep in mind that continued observation is necessary as the endometrial cells may preserve their neoplastic capacity. Women who respond positively should remain protected for years with a long-acting hormone-releasing intrauterine system.
